# Effects of Conventional Heating on the Stability of Major Olive Oil Phenolic Compounds by Tandem Mass Spectrometry and Isotope Dilution Assay

**DOI:** 10.3390/molecules15128734

**Published:** 2010-12-01

**Authors:** Mohamed Attya, Hicham Benabdelkamel, Enzo Perri, Anna Russo, Giovanni Sindona

**Affiliations:** 1 Department of Chemistry, University of Calabria, I-87036 Arcavacata di Rende (CS), Italy; 2 Research Center for Oliviculture and Olive Industry, I-87036 Rende, Italy

**Keywords:** nutraceuticals, olive oil, thermal degradation, isotope dilution assay

## Abstract

The quality of olive oils is sensorially tested by accurate and well established methods. It enables the classification of the pressed oils into the classes of extra virgin oil, virgin oil and lampant oil. Nonetheless, it would be convenient to have analytical methods for screening oils or supporting sensorial analysis using a reliable independent approach based on exploitation of mass spectrometric methodologies. A number of methods have been proposed to evaluate deficiencies of extra virgin olive oils resulting from inappropriate technological treatments, such as high or low temperature deodoration, and home cooking processes. The quality and nutraceutical value of extra virgin olive oil (EVOO) can be related to the antioxidant property of its phenolic compounds. Olive oil is a source of at least 30 phenolic compounds, such as oleuropein, oleocanthal, hydroxytyrosol, and tyrosol, all acting as strong antioxidants, radical scavengers and NSAI-like drugs. We now report the efficacy of MRM tandem mass spectrometry, assisted by the isotope dilution assay, in the evaluation of the thermal stability of selected active principles of extra virgin olive oil.

## 1. Introduction

The beneficial effects of the Mediterranean diet can be attributed not only to the high relationship between unsaturated and saturated fatty acids of olive oil, but also to the antioxidant and anti-inflammatory property of its phenolic compounds. The major phenolic compounds, tyrosol, hydroxytyrosol, oleuropein, and oleocanthal, which give extra-virgin olive oil its bitter, pungent taste, have powerful antioxidant activity both *in vivo* and *in vitro*. Olive oil phenols have also been beneficially linked to processes that contribute to the pathogenesis of heart disease and cancer [1]. In particular hydroxytyrosol, one of the major phenolic constituent in olive oil, has been reported to alone reduce the risk of coronary heart disease and atherosclerosis [2,3], also oleocanthal has been found to have anti-inflammatory and antioxidant properties [4]. Virgin olive oil contains simple and complex phenolic compounds in amounts ranging between 50 and 1,000 mg/kg [5], and it was demonstrated that the variability of those main olive oil phenolic compounds can be related to a combination of agronomic and/or technological processes [6]. The relative proportion of this component depends on several factors such as fruit’s variety, location, degree of ripeness [7,8] and extraction procedure [9,10]. The type as well as their availability is therefore an important parameter in evaluating the quality and nutritive value of virgin olive oil. The profiling of changes in simple phenols present in olive oil over 18 months, showed, with respect to the total phenol content, an initial increase of tyrosol and hydroxytyrosol, which disappeared at the end of the period [8]. It is necessary to point out that refined oils do not have a significant content of the improperly called polyphenols. Nowadays, a great number of cheaper EVOOs sold in supermarkets and discount stores are probably illegal blends of EVOOs and DEOs. These virgin olive oils may be consumed raw in toasts, salads and other foodstuffs [11], but often they are also consumed after domestic heating, such as frying, boiling, conventional and microwave heating [11]; these thermal treatments are commonly utilized for home cooking, food catering and industrial processes [12,13]. 

Most foods are unstable in the thermodynamic sense, this means that they have the tendency to change from a low-entropy (ΔS), and high enthalpy (ΔH) state to a high-entropy, and low enthalpy state.[14,15] Food technology is in fact a battle against this thermodynamic instability. Models that are used in quality change assay are therefore kinetic approaches, describing either the degradation of compounds (such as nutrient content), or their help in controlling and predicting food quality attributes and their changes. The kinetic assessment implies that changes can be captured in mathematical models containing characteristic kinetic parameters, such (reaction order , reaction rate K, and activation energies (Ea), enthalpy (ΔH), entropy (ΔS), and Gibbs free energy (ΔG), Using mathematical models of Eyring (1), Gibbs (2), and Arrhenius:

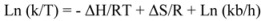
(1)

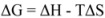
(2)

A multitask effort is needed therefore to fully characterize a functional food such as olive oil in all possible different environments to proper evaluate its healthy effects and to provide consumers and producers with reliable tools for assessing quality and safety of the aliment.[16,17]. In this respect mass spectrometry (MS) plays a perfect role. What is important is to note that the information stored in natural complex matrices, such as olive oil, can be revealed by an extensive use of the proper tools of multistage MS, hyphenated with chromatographic devices, and supported by the isotope dilution method. The effects of exposure to thermal stress and, consequently, to possible chemical modification of the phenolic compounds in olive oil can be traced at the very low ppb range. In the following discussion experimental results will be presented matching the effect of thermal stress on some antioxidant and anti-inflammatory active principles present in low quantity in olive oil. The kinetic study and characterization of the thermodynamic parameters governing the thermo degradation reactions of oleopentanedialdheydes in EVOO will be exploited for the future, to establish kinetics models enabling the prediction of the degradation of such anti-inflammatory drug during EVOO heating and storage.

## 2. Results and Discussion

A comprehensive evaluation of the manufacturing processes on the composition of the volatile and phenolic compounds has been recently published [18]. In our approach, quality assessment can be carried out through the evaluation of selected markers whose thermal alteration may affect the quality of virgin olive oil. Oleuropein (OLP) was chosen as reference compound since it can be considered a marker of good practise in olive oil production [19,20] as well as four of the main phenolics compounds, such as two classic antioxidants, tyrosol (TYR) and hydroxytyrosol (HTYR) and two anti inflammatory active principles, the oleo-pentanedialdehydes (TYROLPD) ([2-(4-hydroxyphenyl) ethyl (3*S*,4*E*)-4-formyl-3-(2-oxoethyl)hex-4-enoate)], also known as oleocanthal [4] and (HTYR-OLPD) [2-(3,4-hydroxyphenyl)ethyl (3*S*,4*E*)-4-formyl-3-(2-oxoethyl)hex-4-enoate]. HTYR-OLPD whose presence in olive oil was already reported [21], has been recently synthesized by a bio mimetic approach and tested with success on primary human vascular endothelial cells [22,23]. It was recently reported the structural behavior of those antioxidant phenols and some of their metabolites by Raman spectroscopy in olive oil [24].

### 2.1. Degradation of TYROLPD, HTYR-OLPD, tyrosol (TYR), and hydroxytyrosol (HTYR)

**Scheme 1:**
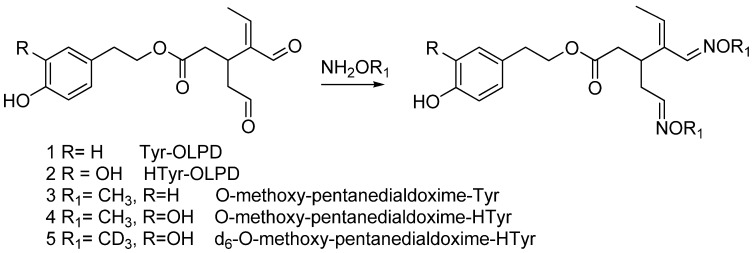
The structure of TYROLPD and HTYROLPD and their labelled and unlabelled O-methyl-hydroxylaminee drivatives.

**Figure 1:**
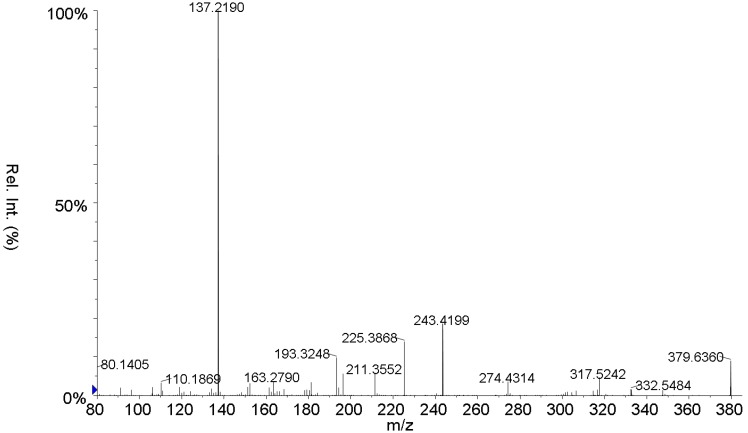
MS/MS spectrum of the protonated molecular ion of O-methoxy-pentanedialdoxime-HTyr.

In this work we wanted to exploit the use of the isotope dilution assay and MRM tandem mass spectrometry for the determination of the residue of the natural ingredients present in micro amounts in different thermally treated oil samples. The case of OLPD species will be discussed in detail. The O-methyl hydroxylamine derivatives, labelled and unlabelled, of the dialdehydes **1**, **2**, among the most important present in olive oil (Scheme 1) can be easily prepared. Compound **1** thus derivatized, provides the ESI spectrum shown in Figure 1. The formation of the *m/z* 137 peak is common to all isotopomers (**1**-**5**). The labelled O-methyl hydroxylamine derivative of compound **2** (*d_6_*-*O*-methoxy-pentanedialdoxime-HTyr, **5**, Scheme 1) was used for the assay of the unlabelled O-methyl hydroxylamine derivatives **1** and **2** by the isotope dilution method with high accuracy and sensitivity. The analytical procedure is very simple since it requires the direct functionalization of the OLPDs present in the aliment preliminary spiked with the labelled reference compound **2** [25].

**Scheme 2:**
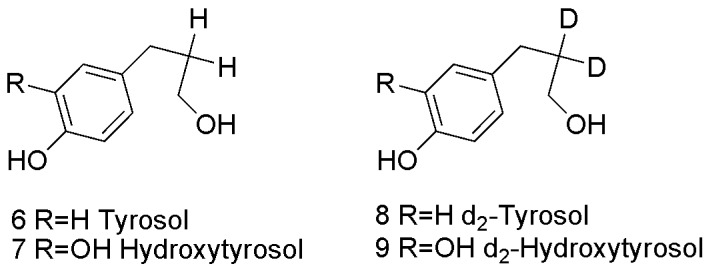
The structure of tyrosol, hydroxytyrosol, d_2_-tyrosol, and d_2_-hydroxytyrosol.

**Figure 2:**
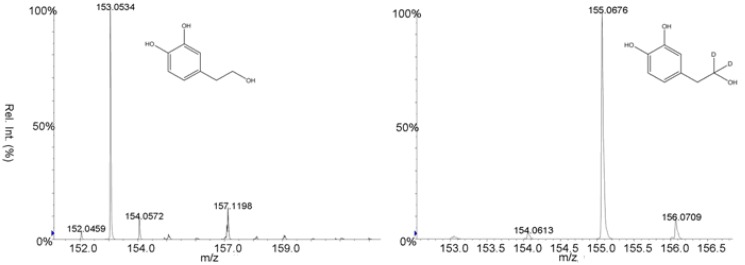
MS/MS spectrum of the protonated molecular ion of hydroxytyrosol and d_2_-hydroxytyrosol.

The determination of the tyrosol residue (TYR, **6**) and hydroxytyrosol (HTYR, 7) (Scheme 2) was carried out using a new developed method by the using of d_2_-tyrosol and d_2_-hydroxytyrosol **8** and **9** (Scheme 2) as internal standards. The method here presented is able to determine trace amounts of those compounds at the ng/g level and share the properties of an accurate assay, being the losses compensated by the use d_2_-tyrosol and d_2_-hydroxytyrosol as internal standards, and of an enhanced specificity (Figure 2).

The methods were extended to the quantitative assay of the other micro components, being aware of the variation in the relative amount of markers in a given oil sample. From the data obtained from two different samples [26] it appears clearly that either the absolute or the relative percentage of the marker content show a significant variation. The effect of temperature was therefore verified for selected samples. 

The kinetic of thermal decomposition of the two anti inflammatory active principles show that for temperature below 100°C the integrity of this molecule is preserved, being the HTYR-OLPD the more stable (Table 1). The complete decomposition of both of them is noticed after about 150 min at 220 °C, also they could not survive more than 150 min at 170°C (Table 1). The same low stability was shown by hydroxytyrosol, since it was no longer present after 150 min at 220 °C (Table 1). Tyrosol showed more stability than all the other phenols, and more than 50% could be presented after heating at 150 °C for 150 min (Table 1). The low stability of TyrOLPD, HtyrOLPD, and HTYR can be explained by the presence of catechol moiety and also by the presence of others active groups which can undergo many side reactions by temperature rising, such as ring closer, polymerization, or hydrolysis in the presence of the ester moiety. The presence of tyrosol could be due to its high stability with referring to the pool of the other phenolic compounds.

**Table 1:** % of TYROLPD, HTYROLPD, TYR, and HTYR losses in EVOO (Oil GABRO 4) and (Oil Bio) heated at 90 °C, 170 °C, and 220 °C.

Oil type	Time (min)	90 °C	170 °C	220 °C
TYROLPD	30	0.77 %	46 %	67 %
	90	18.5 %	53 %	76 %
(Gabro 4 oil)	150	23 %	71 %	84 %
TYROLPD	30	12 %	13.6 %	22.5 %
	90	30.5 %	25.6 %	38.5 %
(Organic oil)	150	38.2 %	66.4 %	99.5 %
HTYROLPD	30	0.6 %	54 %	68 %
	90	1 %	76 %	89 %
(Gabro 4 oil)	150	2.6 %	95 %	97 %
HTYROLPD	30	27 %	53 %	37 %
	90	43 %	85 %	54 %
(Organic oil)	150	52 %	98 %	99 %
Tyrosol	30	0.41%	4%	11%
	90	5%	11%	23%
(Gabro 4 oil)	150	16%	19%	44%
Tyrosol	30	2%	7%	13%
	90	10%	16%	27%
(Organic oil)	150	21%	29%	47%
Hydroxytyrosol	30	5%	14%	23%
	90	31%	36%	61%
(Gabro 4 oil)	150	57%	76%	96%
Hydroxytyrosol	30	7%	15%	27%
	90	30%	34%	66%
(Organic oil)	150	48%	71%	99%

### 2.2. Kinetic studies of the degradation of TYROLPD, HTYR-OLPD

It has been found that food degradation follow the first order kinetics [27,28,29]. Our results show that the degradation upon heating of TYROLPD and HTyr-OLPD at the selected temperature set follow first order kinetics too (Table 2) with specific rate constants (k) for Tyr-OLPD and HTyr-OLPD degradation, as reported in (Table 2).

**Table 2:** The reaction rate constants (k) for Tyr-OLPD and HTyr-OLPD.

	90 °C		170 °C		220 °C	
	k(s^-1^)	R	k (s^-1^)	R	k (s^-1^)	R
TYROLPD	0.066	0.99	0.39	0.96	0.60	0.93
(Gabro 4 oil)						
HTYROLPD	0.01	0.98	0.99	0.97	1.33	0.99
(Gabro 4 oil)						

The activation energy values varied with the type of olive oil samples. The values were 8.9 kJ/mol and 20.1 kJ/mol for degradation of TyrOLPD and HTyr-OLPD respectively in Gabro 4 oil sample; 5.88 kJ/mol and 6.7 kJ/mol for degradation of TyrOLPD and HTyr-OLPD respectively in the Organic oil sample. Lower activation energy implies that a higher temperature change is needed to degrade a specific compound more rapidly. The activation enthalpy (ΔH) and entropy (ΔS) for Tyr-OLPD and HTyr-OLPD showed a similar behavior. The values of ΔH and ΔS were as indicated in Table 3. In general ΔH is a measure of energy barrier that must be overcome by reacting molecules and is related either to the strength of the bonds broken and formed in the formation of the transition state from the reactants and in the solvation effect which may differ for a given molecule, for example a triglyceride, with the environment represented by the constituents of the type of oil being examined. ΔS is related to the number of molecules with appropriate energy that can actually react. The ΔH values were closer to Ea values in the degradation of both TyrOLPD and HTyr-OLPD. In all cases the enthalpy values are always positive, as are the Gibbs free energy values (ΔG); on the other hand, the values of TΔS are always negative, making the reactions no spontaneous. Therefore high temperature is needed for degradation of the examined molecules.

**Table 3:** The activation energy, Ea^*^, the activation enthalpy (ΔH) and entropy (ΔS) for Tyr-OLPD and HTyr-OLPD.

	ΔS J(mol.K)	ΔH (KJ/mol)	Ea* (KJ/mol)	ΔG (KJ)	
TYROLPD	-117	6.3	8.9	90°C	41.7
				170°C	53.9
(Gabro 4 oil)				220°C	63.9
HTYROLPD	-92.2	17.8	20.1	90°C	44.0
				170°C	55.4
(Gabro 4 oil)				220°C	63.4

### 2.3. Degradation of Oleuropein (OLP)

**Scheme 3:**
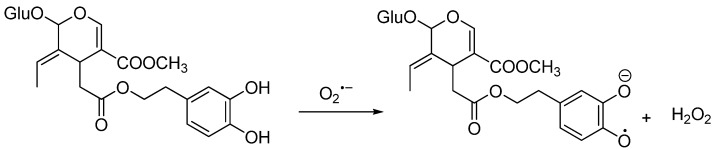
The oxidation of the catechol moiety of the OLP.

A detailed investigation of the thermal effect on OLP was undertaken on the pure analyte dissolved in hexane and in an inert nitrogen atmosphere to avoid possible degradation arising from oxidation of the catechol moiety of the molecule, whose mechanism was thoroughly demonstrated (Scheme 3) [30], or to the action of lipase [31]. OLP is present in filtered and non filtered olive oil on the order of a few ppb [20]. A 10 ppb hexane solution of pure OLP was left at 80°C and 230°C for a sufficient period of time, and its assay was performed by the isotope dilution method and tandem mass spectrometry, using d_3_-OLP as internal standard [19]. The MS/MS spectra of the [M+NH_4_]^+^ ions of both OLP and its d_3_-isotopomer displayed a common fragment at *m/z* 137, which was formed independently of the label position.

**Table 4:** The degradation of OLP from 0 to72 hours at 80 and 230 °C.

Heating temperature	Concentration after hours of heating (ppb)								
	0	1	3	6	9	12	24	48	72
80 °C	10.00	8.62	7.12	5.08	2.76	1.23	0.32	1.114	-^a^
230°C	10.00	0.732	-	-	-	-	-	-	-

^a^ The concentration was less than 0.1 ppb (out of the calibration curve).

At the lower temperature the concentration of the molecule was reduced to half after 6 hours, whereas it was no longer present after 3 hours at 230 °C (Table 4). The thermal effect on OLP was clearly evident after one hour at low and high temperature, and OLP had completely disappeared after 3 hours at 230°C, whereas traces of it could be detected at 80°C for not more than 24 hrs. This approach could be used to verify the loss in the nutraceutical property of EVOOs through the disappearance of OLP in cases of thermal treatments as for high or low temperature deodoration. 

## 3. Experimental

### 3.1. Instrumental and Mass Spectrometry

Mass spectrometric analysis was performed on a triple quadrupole mass analyzer fitted with a heated electrospray ionization (HESI II) source operating in positive ion mode. The following working conditions were applied: spray voltage: 3.5 kV; vaporizer and capillary temperature: 370 and 300 °C, respectively; sheath and auxiliary gas at 40 and 20 arbitrary units (a.u.), respectively; The collision gas was Argon used at a pressure in the collision cell (Q2) of 1.5 mTorr and the mass resolution at the first (Q1) and third (Q3) quadrupole was set at 0.7 Da at full width at half maximum (FWHM). S-lens RF amplitude was kept at 103 V while collision energy (CE) were optimized individually per compound. Selected reaction monitoring (SRM) mode, was used to quantify the analytes: the assay of oleopentanedialdehydes was performed following two transitions per compound: the first one for quantification and the second for confirmation; in particular for 1 the transitions m/z 379 > m/z 137 (assay, CE = 27 eV) and m/z 379 > m/z 274 (confirmation CE = 22 eV), while for compound 2 were used the reactions m/z 363 > m/z 121 (assay CE = 27 eV) and m/z 363 > m/z 258 (confirmation CE = 22 eV). Instrument control and data processing were carried out by means of Xcalibur Software (Thermo Electron, San José, USA). The total LC-MS/MS method run time was 15 minutes. 

High-resolution electrospray ionization (ESI) experiments were carried out in a hybrid Q-Star Pulsar-i (MSD Sciex Applied Biosystem, Toronto, Canada) mass spectrometer equipped with an ion spray ionization source. Samples were introduced by direct infusion (3 μL/ min) of the sample containing the analyte (5 ppm), dissolved in a solution of 0.1% acetic acid, acetonitrile/water 50:50 at the optimum ion spray (IS) voltage of 4800 V. The source nitrogen (GS1) and the curtain gas (CUR) flows were set at pressures of 20 and 25 psi, respectively, whereas the first declustering potential (DP1), the focusing potential (FP), and the second declustering potential (DP2) were kept at 50, 220, and 10 V relative to ground, respectively.

### 3.2. Analytical methods and materials

All the analaytes were analyzed using the isotope dilution assay and MRM tandem mass spectrometry. Oleo-pentanedialdehydes (TYROLPD) ( [2-(4-hydroxyphenyl) ethyl (3*S*,4*E*)-4-formyl-3-(2-oxoethyl)hex-4-enoate)], oleocanthal and (HTYR-OLPD) [2-(3,4-hydroxyphenyl) ethyl (3*S*,4*E*)-4-formyl-3-(2-oxoethyl)hex-4-enoate] were analyzed in oil samples following a previous published method [25], by using The labelled O-methyl hydroxylamine derivatives, as internal standard for the unlabelled *O*-methyl hydroxylamine derivatives (Scheme 1). Oleuropein (OLP) was analyzed as published before [19] as a pure analayte in free oxygen hexane, using d_3_-oleuropein as internal standard (FIG. A). Tyrosol (TYR) and hydroxytyrosol (HTYR) were analyzed using a developed method in which d_2_-tyrosol and d_2_-hydroxytyrosol were used as internal standard. All solvents and chemicals used for analysis and synthesis are commercial available. OLP, TYROLPD, and HTYROLPD were obtained by extraction from olive leaves or synthesized as explained in reference [23]. All the labeled internal standards were synthesized in our laboratory as it was published before. D_2_-tyrosol and D_2_-hydroxytyrosol were synthesized as it was reported in reference [32]. EVOO Samples provided by Gabro Oil [25].

### 3.3. Calibration curves

The calibration curves were built by five points for every analyte, which can be convenient to our analysis, rang. It showed good linearity for all the analytes as were reported in the previous published methods. 

### 3.4. Kinetic data analysis

Nutrient destruction is described using the reaction rate and the dependence of reaction rate on temperature. Parameters used are the reaction rate constant (k) and the Arrhenius activation energy (Ea). The kinetic data were analyzed as described by Van Boekel4. The degradation loss is generally considered to follow first order kinetics:

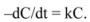
(1)
where C is the concentration of nutrient, t is time and k is the reaction constant (time-1). If C_0_ is the concentration of the Oleopentanedialdheydes at time zero, integration of the Eq. (1) yields:

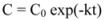
(2)

Taking natural logarithms, we have:

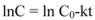
(3)

For the first order reaction, a plot of lnC against time t will be a straight line, and the rate constant is represented by the slope. The rate constant values for the degradation of oleopentanedialdheydes are reported in Table 2. Temperature dependence of a reaction is described by the Arrhenius equation:

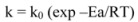
(4)
where, k_0_ = frequency factor or the Arrhenius constant (time^-1^), R is the universal gas constant (8.3145 J/ molK), and T is the absolute temperature (K). Substituting for k from Eq. (5) in (4) 


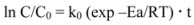
(5)

Enthalpy (ΔH) and entropy (ΔS) of activation were obtained by the regression analysis of ln(k) on 1/T from the equation derived from transition state theory 5:

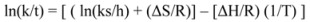
(6)
where ks is Boltzmann constant (1.380658E-23 JK^-1^), h is Plank’s constant (6.6260755E-34Js) and R is universal gas constant (8.3145 Jmol^-1^K^-1^).

### 3.5. Statistical analysis

Thermal stability tests were performed in duplicate, however, all chemical and instrumental measurements were performed in triplicate and the data presented in this work are the mean value of the different experiments. The data were analyzed and the Kinetic parameters were determined using (Microsoft Excel) software (Version 5, Microsoft Corporation, and Redmond, WA, USA).

### 3.6. The thermal treatment of Oleuropein (OLP)

To a dry tube, was added 5 mL of the solution of 10 ppb oleuropein in hexane under N_2_ gas, and the solution was heated at 80 °C for (6, 12, 24, 48, 72 hr), and at 230 for (1, 3, 6, 9, 12, 24 hr), in separate experiments. After the exact time for every experiment, the solution was evaporated to dryness and then solubilised in 500 µL methanol containing 1ppb d_3_-OLP, and it was injected to the instrument.

### 3.7. The thermal treatment of tyrosol (TYR), hydroxytyrosol (HTYR), oleo-pentanedialdehydes (TYROLPD), and (HTYR-OLPD).

Olive oil samples were heated at different temperatures (90, 170, 220°C) for 0–150 min. Approximately 20-g portions of olive oil were placed in a glass beaker, covered with a lid, and heated in a temperature controlled oil bath maintained at selected temperatures (90, 170, 220°C) and were thoroughly mixed to ensure uniform temperature in the sample. After the olive oil attained the desired temperature (± 0.5°C), test beakers were removed from the oil bath at predetermined time intervals (0, 30, 90, and 150 min, after come-up) and transferred immediately to ambient temperature to order to quantitative analyze.

### 3.8. Derivatization procedure for TyrOLPD and HTyrOLPD

Standard solution of **1** at (100 μL of 1,000 mg/L) were added to a 1.5 M solution of *O*-methylhydroxylammonium chloride (1 mL) in methanol and heated at 55°C for 60 min. Finally the mixture containing the *O*-methoxy-pentanedialdoxime-HTyr (**4**) was cooled and centrifuged at 4000 rpm for 5 min and diluted ten times with CH_3_OH/H_2_O 70/30. Analogously, a stock solution of the *d_6_*-*O*-methoxy-pentanedialdoxime-HTyr (**5**) was prepared using d_3_-O-methylhydroxylammonium as derivatizing agent. 

Sample preparation: olive oil (100 mg) was mixed with a 1.5 M solution of *O-*methylhydroxylammonium chloride (900 μL). The mixture was stirred at 55 °C for 60 min; after cooling, the reaction mixture was centrifuged at 8,000 rpm for 1 min and then supernatant (100 μL) was mixed with 10 mg/L of the internal standard dissolved in 70:30 CH_3_OH/H_2_O (100 μL). The mixture were diluted to 1 mL with 70:30 CH_3_OH/H_2_O solution; the resulting solution was thoroughly mixed twice by vortexing for 30 s to allow homogeneous distribution of the standards; at the end the mixture was diluted (1/10), filtered through a 0.22 μm and then injected into the instrument.

### 3.9. Sample preparation of tyrosol (TYR), hydroxytyrosol (HTYR)

Olive oil (100 mg) was mixed with the internal standard d_2_-tyrosol or d_2_-hydroxytyrosol dissolved in CH_3_OH/H_2_O 70/30 (100 μL of 10 mg/L solution). The mixture were diluted to 1 mL with a 70:30 solution of CH_3_OH/H_2_O; the resulting solution was thoroughly mixed twice by vortexing for 30 s to allow homogeneous distribution of the standards; at the end the mixture was diluted (1/10), filtered through a 0.22 μm and then injected into the instrument.

## 4. Conclusions

An unconventional approach to the evaluation of thermal stability of nutraceuticals content of extra virgin olive oil has been presented. The novelty of the present approach is represented by the extensive use of hyphenated mass spectrometric methodologies based on multistep ion analysis. The thermal stability of the most abundant NSAIDs (Non-steroidal anti-inflammatory drugs) has been checked for the first time and new insights into the thermodynamic and kinetic parameters or active principles degradation *versus* temperature/time have been evaluated. The method can be used to trace fraudulent processed olive oils, such as its application to the detection of fraud practices particularly to EVOO mixed with DEOO. 
